# *In situ* assembly of Ag nanoparticles (AgNPs) on porous silkworm cocoon-based wound film: enhanced antimicrobial and wound healing activity

**DOI:** 10.1038/s41598-017-02270-6

**Published:** 2017-05-18

**Authors:** Kun Yu, Fei Lu, Qing Li, Honglei Chen, Bitao Lu, Jiawei Liu, Zhiquan Li, Fangying Dai, Dayang Wu, Guangqian Lan

**Affiliations:** 1 College of Textile and Garments, Southwest University, Chongqing, 400715 China; 2 Chongqing Engineering Research Center of Biomaterial Fiber and Modern Textile, Chongqing, 400715 China; 3 The Ninth People’s Hospital of Chongqing, Chongqing, 400700 China

## Abstract

Preventing wound infection and retaining an appropriate level of moisture around wounds represent the most critical issues in wound treatment. Towards these ends, special focus has been placed on *Bombyx mori* cocoons because the protective function of the silkworm cocoon resembles the manner in which the skin protects the human body. We have designed a facile technique to develop a novel silkworm cocoon-based wound film (SCWF) wound dressing utilizing a CaCl_2_-ethanol-H_2_O solution. To improve the anti-bacterial performance of SCWF, we have incorporated the ability of silk sericin to act as a reducing agent for the conversion of Ag^+^ to Ag, yielding nanoparticles (AgNPs) linked together by peptide bonds of silkworm cocoon wound film (SCWF-AgNPs). SCWF-AgNP dressing exhibited excellent biocompatibility, anti-bacterial performance, and good extensibility. Furthermore, *in vivo* experiments indicated that SCWF-AgNP dressing was able to significantly accelerate the healing rate of infected wounds in New Zealand White rabbits and histological examination revealed that it aided in the successful reconstruction of intact and thickened epidermis during 14 days of healing of impaired wound tissue. These results demonstrate that the present approach might shed new light on the design of anti-bacterial materials such as SCWF-AgNPs with promising applications in wound dressing.

## Introduction

Bacterial infection represents one of the most critical issues in skin injuries. Specifically, skin injury leads to a defect in one of the primary functions of the skin; i.e., a loss of the physical barrier against exogenous microbial invasion, thus rendering the host susceptible to bacterial infection^1^. In turn, the healing process of tissue injuries is complex. As severe wound dehydration could disturb the ideal moist healing environment and delay wound healing^2–6^, advanced antimicrobial and moist wound dressings are therefore witnessing increased demand within the wound care market.

Antibacterial wound dressings have developed very rapidly in recent years, particularly different natural polymer-based engineered wound dressings^7,8^. As a favoured and natural wound dressing material, silk fibroin has been recognized as a biological material owing to its unique properties including excellent biocompatibility, proper mechanical properties, and regenerative performance^9–12^. Silk fibroin film has been successfully produced for the treatment of wounds and such silk films have been shown to heal skin wounds more rapidly than traditional porcine-based wound dressings^13–15^. Generally, electrospinning is considered an attractive method for silk fibroin wound dressing fabrication because electrospun fibre has a large surface area with high porosity^16,17^. For example, electrospun silk fibroin/poly(L-lactide-co-caprolactone) nanofibre membrane can enhance retinal progenitor cell proliferation and differentiation^18^. The functions of biodegradable electrospun silk scaffolds applied as wound dressings have been evaluated^19^; however, these electrospun nanofibre retain challenges, such as bioavailability and high product cost^17^. In particular, production of these electrospun silk scaffolds requires an extremely complex technical and production process, with a series of processing steps being required as silk fibroin cannot be acquired directly but must be extracted by dissolving the degummed silk^20^. These steps include the removal of sericin and the dissolving of silk proteins as well as the bioactive functional proteins remaining from the folding process^20,21^. Therefore, it is of high priority to develop a robust and simplified approach to prepare silk protein biomaterials. To address this issue, researchers have paid special attention to *Bombyx mori* cocoons themselves. The silkworm (*B. mori*) cocoon is formed by sericin wrapped around a fibroin structure. The compact structure of the silkworm cocoon provides high mechanical resistance against disturbance, allowing the cocoon to withstand threats of parasites and predators while providing security for pupal growth and development^22–24^. As such, the protective function of the silkworm cocoons resembles the manner in which the skin protects the human body, suggesting that retention of the full cocoon structure with both sericin and fibroin might be beneficial for wound repair.

AgNPs have demonstrated potential use in biomedical applications owing to their wide antibacterial properties^25–27^. In general, AgNP biomaterials have been fabricated mainly through three methods: admixture^28^, surface coating or adsorption^29,30^, and complexing or covalent cross-linking with modified AgNPs^31,32^. However, there are several distinct disadvantages with these methods. For example, it is extremely difficult to obtain uniformly dispersed AgNPs in three-dimensional matrices by using the first two methods, whereas the third method requires complicated chemical processes and separation. In addition, the AgNPs wrapped in matrix would not be released, thus leading to suboptimal antibacterial activity.

In the current study, inspired by the protective functionality of the whole silkworm cocoon, we developed a novel strategy to prepare AgNP silkworm cocoon-based wound films *in situ* and explored their application in the healing of infected wounds. To avoid the aggregation of AgNPs, we exploited recent findings indicating that the aspartic and glutamic acid residues in sericin play an important role in the reduction of Ag^+^ ^33,34^. Thus, sericin-containing porous silkworm cocoon wound film (SCWF) was initially prepared by treating silkworm cocoons in a CaCl_2_-ethanol-H_2_O solution, as it is much easier to achieve homogeneous dispersion of sericin. By subsequently immersing SCWF in AgNO_3_ aqueous solution, the Ag^+^ molecules immobilized in the porous silkworm cocoon wound film were reduced *in situ* to form AgNPs by sericin in the matrix. Via the presence of AgNPs, the designed AgNP silkworm cocoon wound film (SCWF-AgNP) material was also expected to impart antibacterial activity. Through this method, not only was sericin retained as a portion of the cocoon structure but it could also act as a reducing agent for the conversion of Ag^+^ to Ag in the subsequent process. Thus, this method markedly decreases the complexity of the wound film production process and reduces the cost of AgNP preparation as well.

This paper reports the synthesis of SCWF-AgNP composites with different concentrations of AgNPs to SCWF as well the systematic investigation of their structure, mechanical performance, biocompatibility, anti-bacterial activity, and ability for accelerating infected wound healing. In summary, this work attempted to provide a facile pathway for developing anti-bacterial dressings from silkworm cocoon for infected wound healing.

## Results and Discussion

### Preparation of SCWF and SCWF-AgNPs

**Figure 1:**
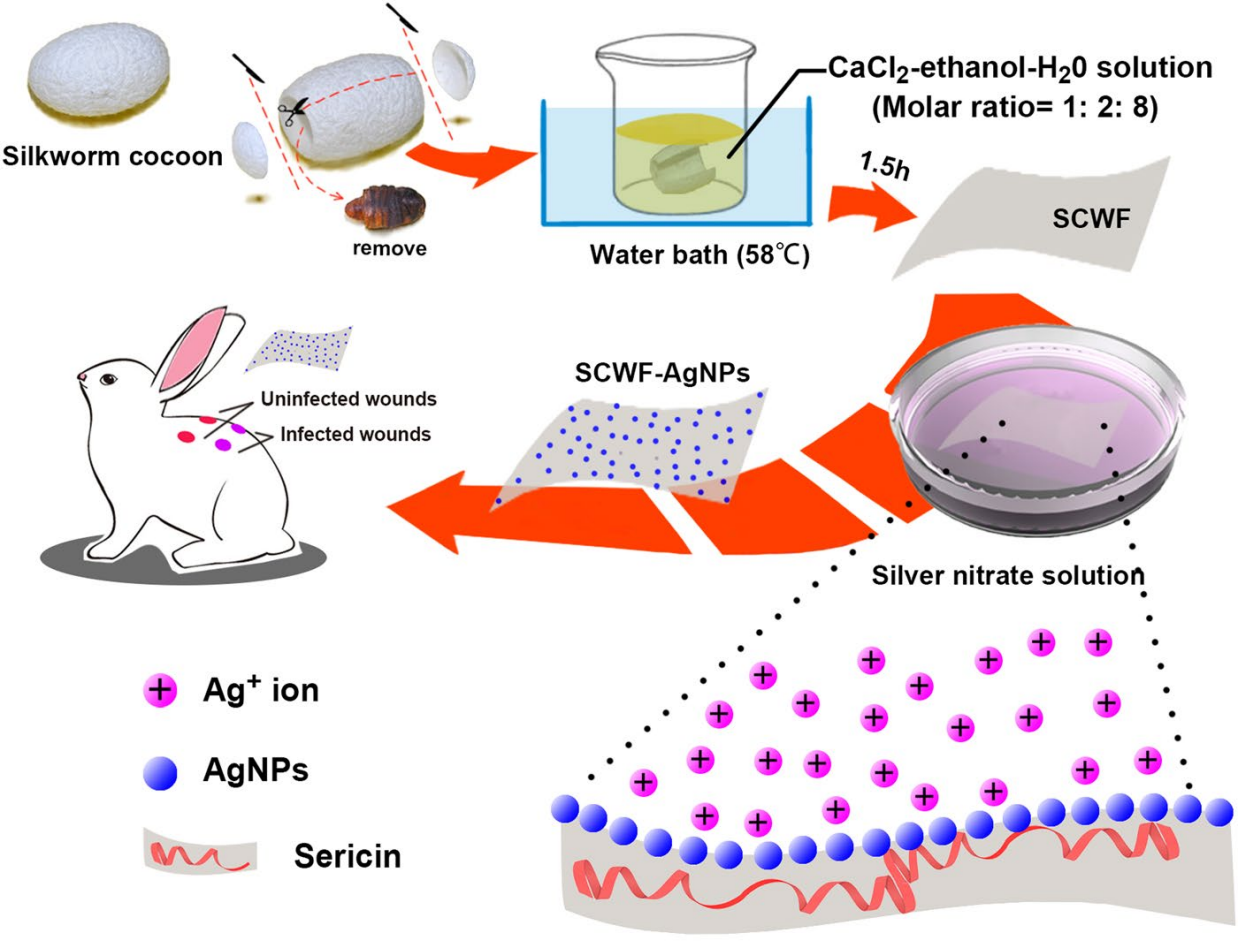
Illustration of the route of SCWF synthesis and the use of SCWF-AgNPs as a wound dressing.

SCWFs were fabricated by the following method (Fig. 1) under constant temperature (20 ± 1 °C) and relative humidity (65 ± 2%) in the laboratory. First, the ends of the cocoon were cut off and the silkworm chrysalises were removed from the cocoons. A solution was prepared containing calcium chloride, ethanol, and H_2_O (molar ratio = 1:2:8). Subsequently, the prepared cocoons were soaked in the CaCl_2_-ethanol-H_2_O solution and the cocoons were incubated for 90 min in a water bath (58 °C) to observe changes in the solution. Once the cocoons became transparent, they were removed and washed 4–5 times with deionized water to obtain the colourless and transparent SCWF samples.

These SCWFs were immersed in an AgNO_3_ aqueous solution to reach equilibrium at room temperature for 4 h. Briefly, 20 mL AgNO_3_ aqueous solution was added to a glass culture dish (diameter 100 mm) and the prepared SCWFs were immersed in the AgNO_3_ aqueous solution after washing 3 times with deionized water. The resultant products were coded as SCWF-Ag1 to SCWF-Ag6 according to the solution concentration of AgNO_3_ (5, 10, 50, 100, 200, and 300 mM, respectively).

The AgNP content in the SCWF-Ag NPs was also determined by UV-visible (UV-vis) spectra, wherein the absorbance of a 0.4 mg/mL solution of SCWF-AgNPs in deionized water was measured using a TU-1901 spectrophotometer (Persee, Beijing, China) across the wavelengths of 800–350 nm.

### Structure and morphology of SCWF and SCWF-AgNPs

**Figure 2:**
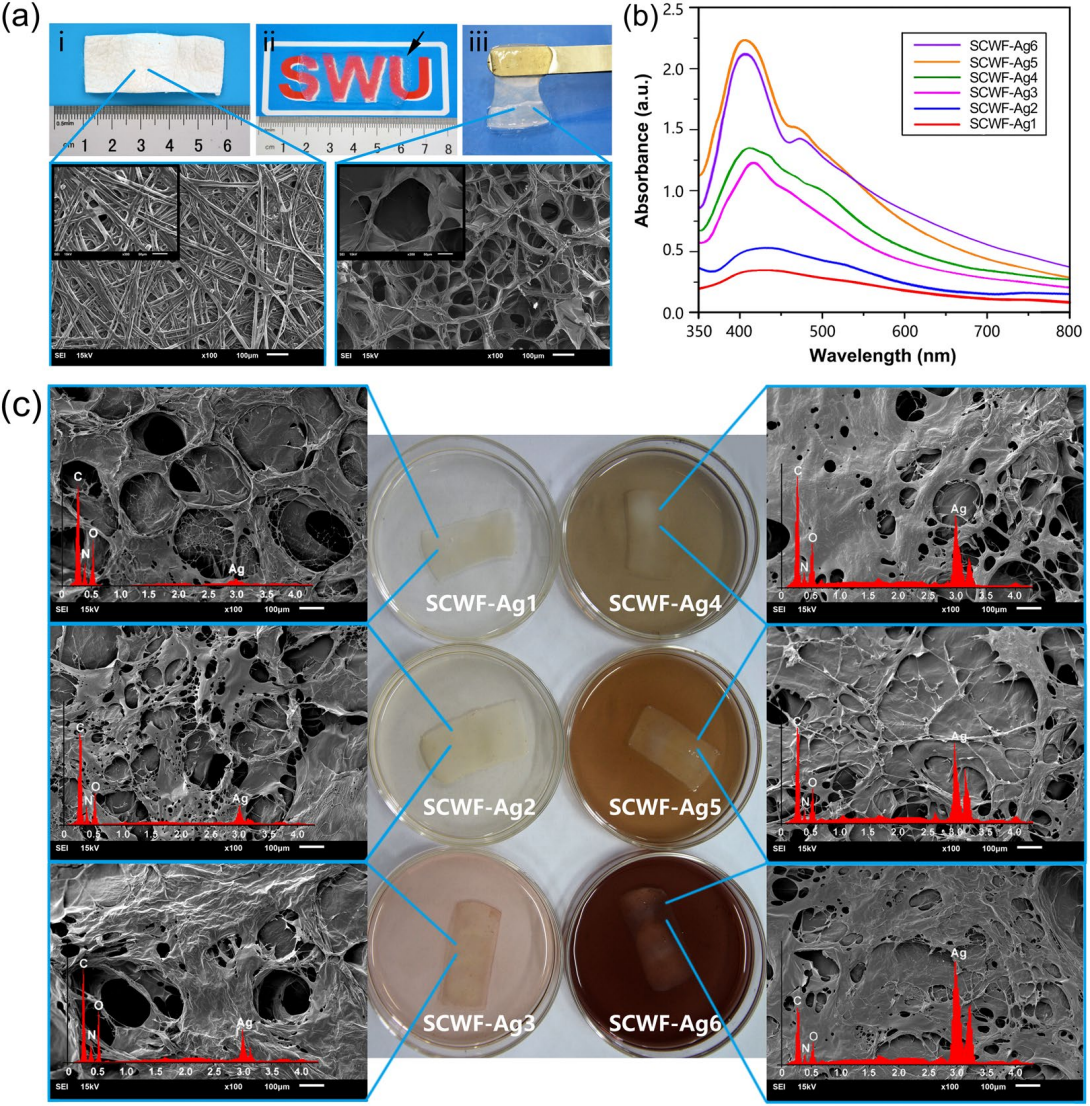
(**a**) Natural *Bombyx mori* cocoons (i); SCWF optical clarity and size (ii), wherein SCWF (black arrow) was placed on a piece of paper with one “SWU” symbol underneath. The gel (balanced on a metal spatula) was sufficiently elastic and flexible for easy handling (iii). The fibroin and sericin further dissolved to form a network that readily aggregated into transparent films, as shown in the scanning electron micrograph of lyophilized SCWF. (**b**) UV-vis absorption spectra of leaching aqueous dispersions of SCWF-Ag1–6 in deionized water. (**c**) Scanning electron micrographs of lyophilized SCWF-Ag1–6 with red curves of EDX and photographs of the respective SCWFs immersed in different concentration of AgNO_3_ aqueous solution after 4 h.

Silkworm cocoons were partially dissolved to form transparent SCWFs. The transparency could be visually observed by the clarity of the symbol “SWU” on a paper underneath the SCWFs (Fig. 2(a)ii). Such optical transparency facilitated wound observation and their material properties enabled painless wound cleaning during the experimental procedures. Further dissolution and condensation of the fibroin and sericin gave rise to dense three-dimensional networks, as shown in the scanning electron micrograph of lyophilized SCWFs (Fig. 2a). Figure 2b presents the UV-vis absorption spectra of the aqueous dispersions of various SCWFs with different AgNP content. The incorporation of AgNPs leads to absorption peaks around 400 nm whose intensity tended to gradually increase with the increase of AgNP content; however, the absorption peak of SCWF-Ag1 was insignificant owing to the extremely low dosage of AgNPs. These absorption peaks of Ag NPs have been attributed to the silver nitrate reduction into AgNPs by the sericin of on the surface of the SCWFs^35^. At higher concentrations of AgNPs, the fibroin and sericin readily aggregate into continuous sheets (Fig. 2c). At low AgNO_3_ concentration, the resulting aqueous dispersions were colourless and transparent; as the AgNO_3_ concentration increased, the aqueous dispersions darkened. The interconnected porous morphology of SCWF was predicted to endow the materials with numerous potential advantages such as absorbing exudates and maintaining a moist environment around the wound interface. The corresponding energy-dispersive X-ray (EDX) spectra of the SCWF-AgNP samples (red curves in Fig. 2c) confirmed the presence of AgNPs. Furthermore, the EDX profile demonstrated a very strong silver signal along with smaller carbon and oxygen peaks, which might have originated from the amidogen of sericin and fibroin bound to the surface of the AgNPs^4,36^. The Ag contents obtained from the EDX spectra of the different samples were consistent with the results of UV (Fig. 2b).

### Mechanical properties of wet SCWF and SCWF-AgNPs

**Figure 3:**
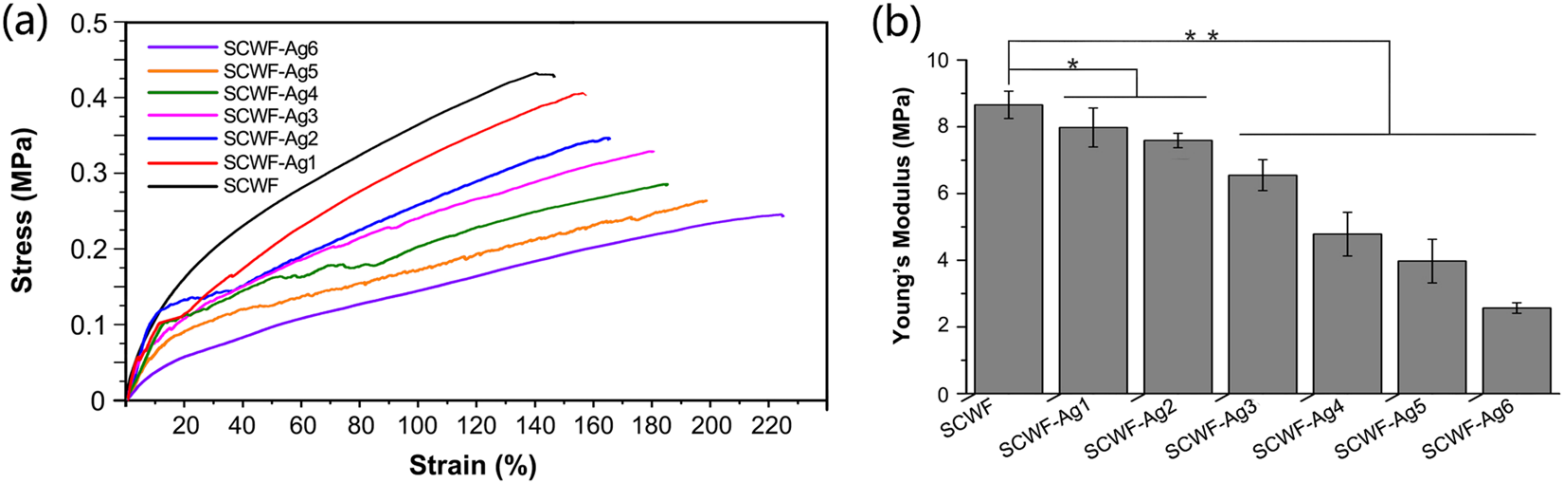
Mechanical properties of SCWF and SCWF-AgNPs. (**a**) Representative strain-stress curves. (**b**) Young’s modulus (*P < 0.05, **P < 0.01, n = 10).

Conventional tensile tests performed on SCWF with and without AgNPs showed that AgNPs exhibited an obvious effect on the mechanical properties of wound dressings (Fig. 3a). Images of the setup used for stretching the sample and measuring its tensile mechanical properties are shown in Supplementary Fig. S1.

AgNPs were able to improve the strain of SCWFs but reduced their strength. Figure 3b shows the Young’s moduli of SCWF and SCWF-AgNPs. In the SCWF-AgNP specimens, as the AgNP content increased, the Young’s modulus decreased from 8.658 ± 0.004 MPa for SCWF to 2.566 ± 0.002 MPa for SCWF-Ag6. The Young’s moduli of SCWF-AgNPs were decreased in comparison with SCWF, with a highly significant negative correlation. These values are comparable to the Young’s modulus of some native tissues such as skin dermis^37^. Similarly, as the AgNP content increased, the ultimate tensile stress decreased from 0.465 ± 0.048 MPa in SCWF to 0.225 ± 0.034 MPa in SCWF-Ag6 (see Supplementary Fig. S2). Notably, the ultimate elongation values all exceeded 140%. As the AgNP content increased, the ultimate elongation increased from 147.0 ± 4.7% in SCWF to 229.3 ± 15.8% in SCWF-Ag6 (see Supplementary Fig. S2). The marked consumption of sericin by Ag^+^ results in a further decrease of the stress, and thus more elasticity. The excellent elasticity of SCWF-AgNPs is expected to enhance wound contact.

### Characterization of SCWF and SCWF-AgNPs

**Figure 4:**
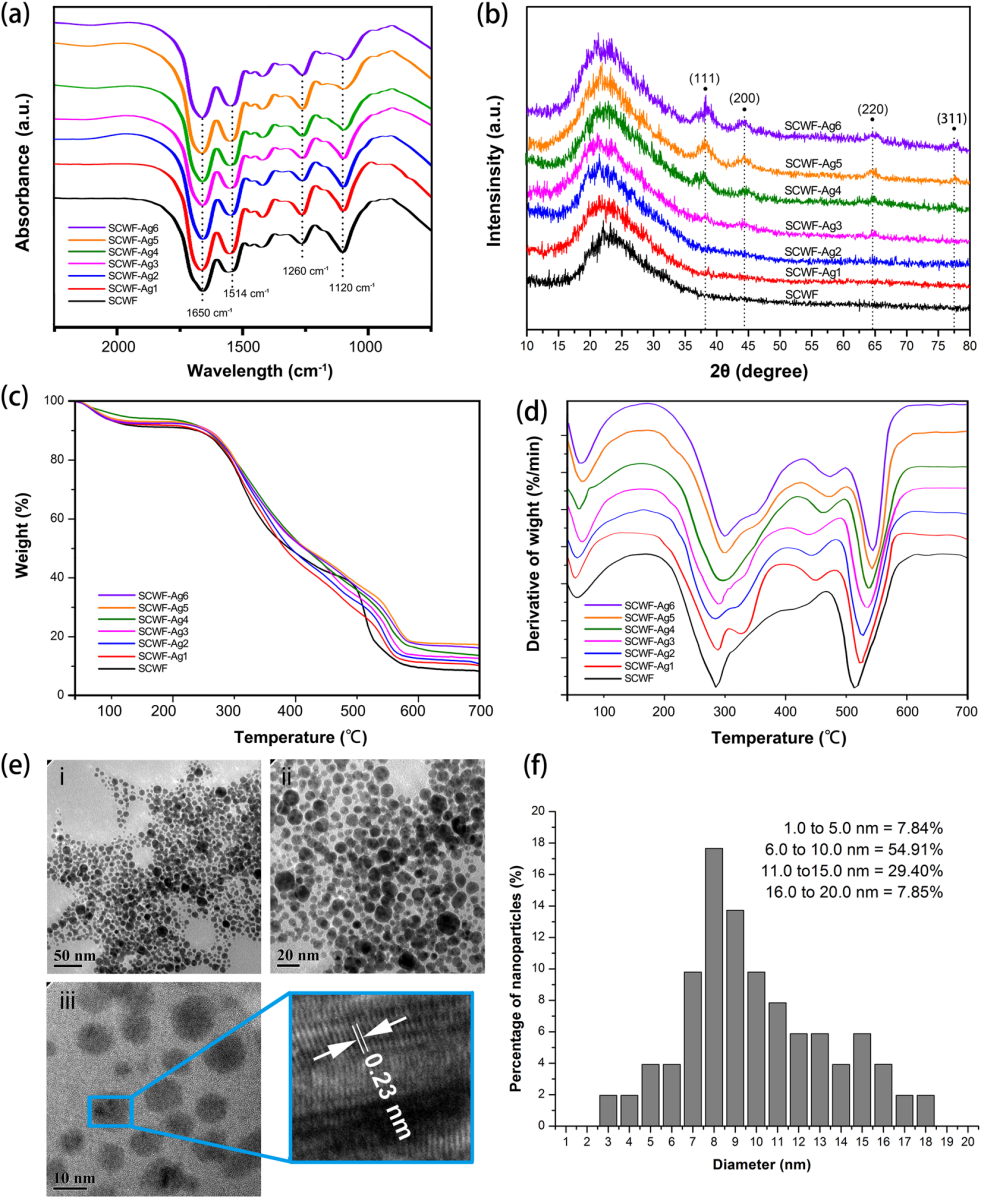
Characterization of SCWF and SCWF-Ag1–6: (**a**) FT-IR absorbance spectra. (**b**) XRD characterization. (**c**) Thermo-gravimetric curves. (**d**) Differential thermal gravity curves. (**e**) TEM images of SCWF-Ag6 at different magnifications of 50 (i), 20 (ii), and 10 nm (iii); (**f**) the corresponding diameter size distribution.

The FTIR spectra of SCWF and SCWF-Ag NPs are shown in Fig. 4a. The infrared spectral region between 1700 and 1500 cm^−1^ is generally utilized for the analysis of different secondary structures of silk fibroin. The peaks at 1610–1630, 1695–1700, and 1510–1520 cm^−1^ are characteristic of the silk II (β-sheet conformation) secondary structure of silk fibroin, whereas the absorptions at 1648–1654 and 1535–1542 cm^−1^ are characteristic of the silk I (α-form) conformation. In Fig. 4a, the peaks at 1620, 1514, and 1238 cm^−1^, representing sericin amide I (C=O stretching), amide II (C–N stretching, N–H deformation), and amide III (C–N stretching), respectively, indicate a random coil/silk I conformation^38^. The peaks at 1620 cm^−1^ indicate C=O stretching of carboxylate ions and at 1120 cm^−1^ show C–O stretching of the phenolic hydroxyl group on the tyrosine of sericin^39^. In a larger view of the spectrum, after the reaction, the absorption intensity can be seen to weaken at 1120 cm^−1^, indicating that the phenolic hydroxy residues had broken off^40^; these phenolic hydroxyl functional groups could be oxidized to form the quinone structures. In addition, after the reaction, the infrared spectrum of SCWF-AgNPs all exhibited an absorption band that related to the nitrogen atom structure red-shift as a result of the nitrogen atoms on the molecular structure of sericin combining with the silver atoms or ions.

Figure 4b shows the X-ray diffraction (XRD) patterns of SCWF and SCWF-AgNPs. SCWF showed a typical diffraction peak at about 23°, corresponding to the diffraction peak of silk fibre^41^. Sericin acts as a reducing agent and dispersant; therefore, upon the addition of SCWF, AgNO_3_ could be reduced by the sericin groups of SCWF to generate AgNPs, as evidenced by the relevant XRD analysis. The small peaks at 2θ = 38.1°, 44.2°, 64.5°, and 77.5° were assigned to the (111), (200), (220), and (311) planes of crystalline Ag (Fig. 4b), and their intensities also depended on the Ag content in the materials^42^. The XRD patterns of SCWF-Ag4, SCWF-Ag5, and SCWF-Ag6 were analysed to confirm the existence of AgNPs.

Figure 4c represents the thermal decomposition curves for SCWF and SCWF-AgNP dressings, which were assessed by thermo-gravimetric analysis. The figure revealed that all of the SCWF and SCWF-AgNP dressings exhibited a thermal degradation temperature between 280 °C and 560 °C, which completely satisfied the required thermal stability of the wound dressing^43^. As shown in Fig. 4d, as the concentration of silver nitrate increased, the thermal degradation temperatures of the materials were higher. The thermal stability of the SCWF dressing with the incorporation of silver was observed to change within the range of 14 °C. Therefore, the incorporation of a small quantity of AgNPs had no obvious effect on the pyrolysis temperature of the composite sponges.

The size and the shape of the NPs are considered very important because of their different physical and chemical properties. TEM is one of the best adapted methods to discover the size and shape of the NPs and determine their distribution^44^. Figure 4e illustrates the AgNPs synthesized using sericin as the reductive agent; the images represent SCWF-Ag6 at different magnifications of 50, 20, and 10 nm, respectively. The AgNPs are quite homogeneously distributed and exhibit an excellent spherical shape. The TEM images confirmed that the particle size of AgNPs ranged from 3 to 18 nm (Fig. 4e). The analysis results also demonstrated that the AgNPs were in metallic form rather than present as Ag compounds.

### Anti-bacterial performance of SCWF-AgNPs

**Figure 5:**
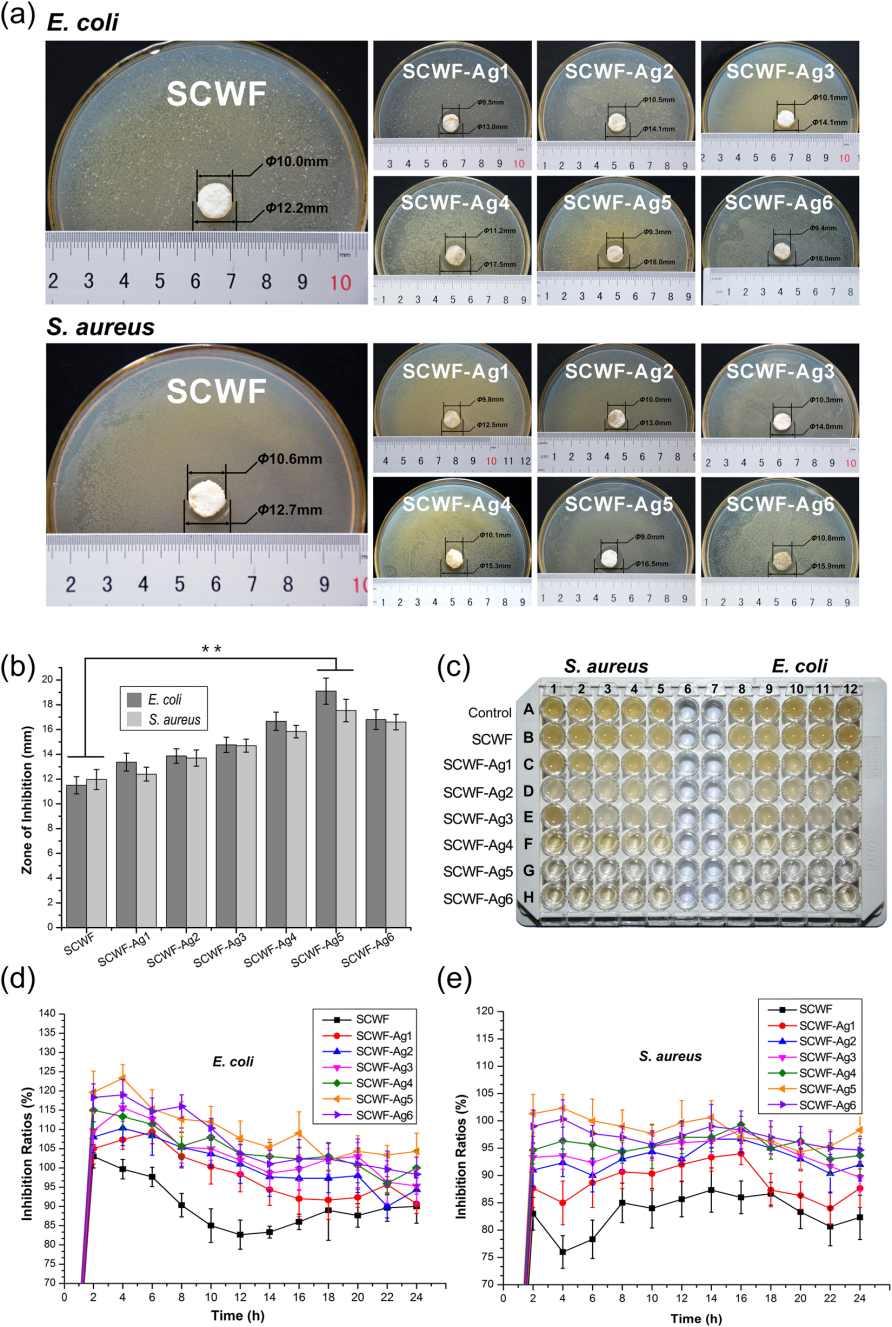
Anti-bacterial performance of SCWF-AgNPs. (**a**) Inhibition zones of SCWF and SCWF-Ag1–6 after 24 h incubation at 37 °C. (**b**) Zone of inhibition diameters (mm) of SCWF/SCWF-AgNP samples against *S. aureus* and *E. coli* after incubation for 24 h (**P < 0.01). (**c**) The 96-well plate image of bacteria in broth media after being directly in contact with SCWF or SCWF-Ag1–6 for 24 h. Inhibition ratio kinetic curves of SCWF and SCWF-Ag1–6 against *S. aureus* (**d**) and *E. coli* (**e**). The error bars denote the standard error of the mean (n = 3).

Figure 5 shows the anti-bacterial performance of SCWF/SCWF-AgNPs against *Escherichia coli* and *Staphylococcus aureus*, which are common bacteria found in wound infections. The inhibition zones of SCWF/SCWF-AgNPs against *E. coli* and *S. aureus* are shown in Fig. 5a. After 24 h incubation at 37 °C, all samples exhibited different degrees of anti-bacterial performance, with the sample of SCWF-Ag5 clearly demonstrating anti-bacterial zones against both *E. coli* (18.0 mm) and *S. aureus* (16.5 mm) (Fig. 5b). The control sample, SCWF, exhibited only very weak inhibition against the two kinds of bacteria. In comparison, the zones of inhibition of SCWF against *E. coli* and *S. aureus* were 12.2 and 12.7 mm, respectively. Images of bacteria in broth media after incubation with SCWF or SCWF-Ag1–6 for 24 h are show in Fig. 5c. The bacterial inhibition effect could be visually observed in the 96-well plate. The bacterial inhibition ratios of SCWF and SCWF-AgNPs against *E. coli* were all higher than 90% after 6 h incubation of 0.2 g freeze-dried sample in 20 mL bacteria suspension (initial OD600 of 0.1) (Fig. 5d), indicating that the contained AgNPs effected very good *E. coli* inhibition. In particular, the bacterial inhibition ratios of SCWF-Ag5 (800 μL of 0.01 g/mL AgNO_3_) against *E. coli* were all higher than 100% during the 24 h of incubation. Although the inhibition ratios of the SCWF-AgNPs against *S. aureus* were not as high as those against *E. coli*, over 80% of *S. aureus* were inhibited at all time points during 0–24 h (Fig. 5e). The differences of SCWF-AgNPs against Gram-negative bacteria *E. coli* and Gram-positive bacteria *S. aureus* could be attributed to the different structure of their cell wall^45^. The *S. aureus* inhibition ratios of SCWF-Ag5 were all approximately 100%, slightly lower than those against *E. coli*. Overall, the SCWF-Ag5 samples exhibited excellent inhibition against *E. coli* and *S. aureus*. However, EDX, XRD and UV absorption results proved that SCWF-Ag6 contains more AgNPs compare to SCWF-Ag5, we analysed the cause of the result was because SCWF-Ag6 contains more AgNPs and agglomeration is caused easily (Fig. 4e). The highly aggregated AgNPs would have a limited inhibition against *E. coli* and *S. aureus*^4^.

### Biocompatibility of SCWF-AgNPs

Cytotoxicity, another criterion to evaluate the feasibility of a dressing for wound healing, is often assessed by the 3-(4,5-dimethyldiazol-2-yl)-2,5-diphenyltetrazolium bromide (MTT) assay, which is commonly used to analyse the possible harmful effects induced in cells by materials^46,47^. The cytotoxicity of SCWF-AgNPs was evaluated by standard MTT assay in murine L929 fibroblasts, which was used as a model cell line. The cells were treated with various leaching liquors from SCWF, SCWF-Ag5 and SCWF-Ag6; the resultant cell viabilities are found as Supplementary Fig. S3. The results indicated no cytotoxicity regardless of the incubation time. Both SCWF and SCWF-Ag5 exhibited high biocompatibility with cell viabilities over 100%. Although the increasing of AgNPs content in SCWF decreased slightly the L929 cell viability, all samples exhibited high biocompatibility, and their cell viabilities were higher than 85%.

**Figure 6:**
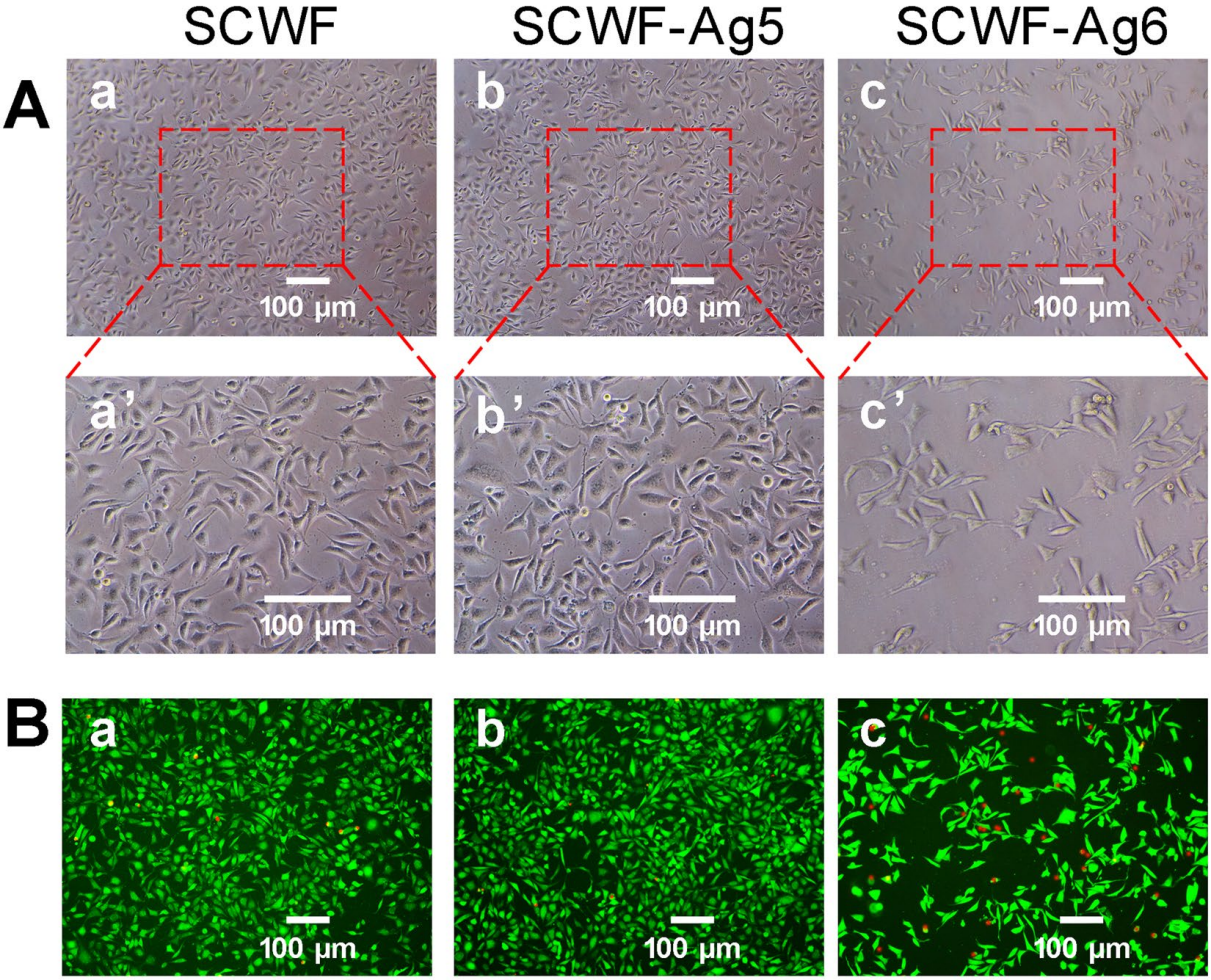
(**A**) Growth observations of L929 cells treated with SCWF (a,a’), SCWF-Ag5 (b,b’), and SCWF-Ag6 (c,c’). (**B**) Calcein-AM/PI Double Stain Kit assay of L929 cells upon treatment with SCWF (a), SCWF-Ag5 (b) and SCWF-Ag6 (c). Live cells are stained by Calcein AM dye and produce an intense uniform green fluorescence (ex/em ~495 nm/~515 nm). Dead cells are stained by Calcein PI dye and emit bright red fluorescence (ex/em ~495 nm/~635 nm). The scale bar represents 100 μm.

SCWFs were beneficial to the growth of L929 murine fibroblast cells in the paste (Fig. 6A). After the L929 cells were treated with SCWF and SCWF-Ag5/6 for 24 h, morphological observations of the L929 cells that proliferated show that the cells were spindle-shaped (Fig. 6A,a–c), and revealed that cells treated with SCWF and SCWF-Ag5 emitted strong green fluorescence accompanied by cellular apoptosis (Fig. 6B,a and b). Moreover, with SCWF and SCWF-Ag5 (Fig. 6B,a and b), the cells appear to be more evenly distributed and organized in clusters. However, the growth of L929 cells treated with the basal medium and SCWF-Ag6 was sparse (Fig. 6B,c and c’). SCWF-Ag6 emitted scattered green fluorescence accompanied by cellular apoptosis (bright red fluorescence). The L929 cell viability of SCWF-Ag6 was lower than that of SCWF and SCWF-Ag5, perhaps to be relative with the presence of the AgNPs clusters, the cytotoxicity of AgNPs elevate with the increase of AgNPs contents^4,48^. These results agree with the MTT results, further supporting that the porous structure of SCWFs support cell activity. Thus, we can conclude that SCWF and SCWF-Ag5 were beneficial to the adhesion of fibroblasts, which is beneficial for wound dressing applications.

### Uninfected- and infected-wound healing efficacy of SCWF-AgNPs

**Figure 7:**
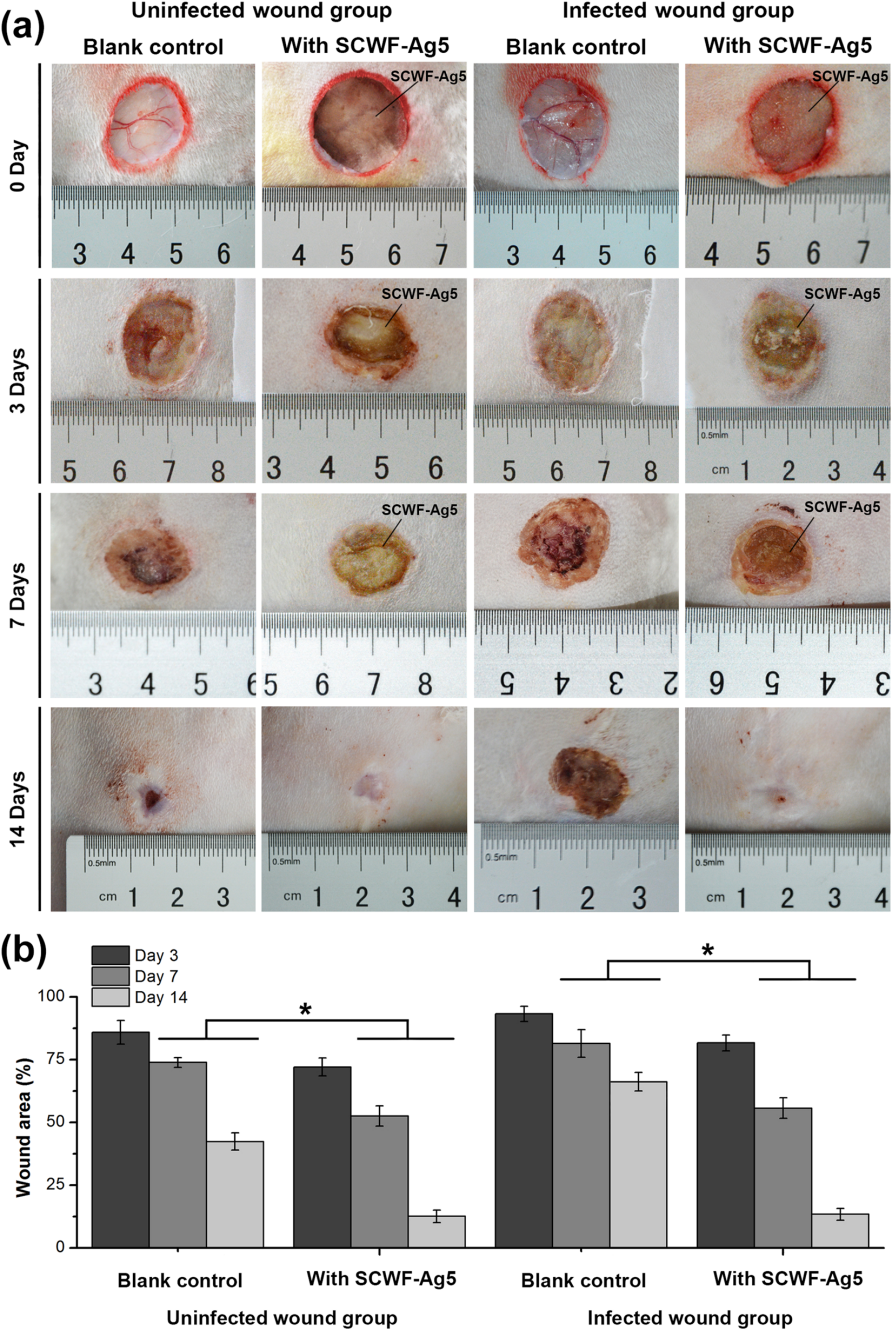
SCWF-Ag5 enhances wound contracture. (**a**) Visual observation of surface healing in the uninfected- and infected-wound groups. SCWF-Ag5 enhanced wound contracture. All the images in this Figure show the progression of wound healing for the same animal. (**b**) Quantitative evaluation of wound healing by measuring wound area when untreated and treated with SCWF-Ag5. Values are the means ± SD for each group (n = 3, *P < 0.05).

Re-epithelialization is important in wound healing as the skin plays a major barrier function in protecting the host against pathogens^49^. Figure 7a shows images of the uninfected- and infected-wound groups treated with SCWF-Ag5 and without dressing for 0, 3, 7, and 14 days. For the uninfected wound group, the blank control exhibited no significant effects on the wound size after 3-day treatments, with the wound area of the uninfected group as 85.93 ± 4.75% (Fig. 7b). However, unlike the blank control, SCWF-Ag5 tested under the same conditions was able to effectively reduce the wound size and inflammation; after 3- and 7-day treatment with SCWF-Ag5, the wound areas of the uninfected group were 72.11 ± 3.59% and 52.55 ± 4.04%, respectively (Fig. 7b). For the infected-wound groups, the infected-wound without dressing exhibited obvious inflammation after 3 and 7 days (Fig. 7a), with wound areas of 93.27 ± 3.06% and 81.48 ± 5.48%, respectively (Fig. 7b). Notably, SCWF-Ag5 was able to effectively reduce the size and inflammation of the infected wound upon 7-day treatment, to 55.71 ± 4.14%. After 14-day treatment with SCWF-Ag5, the dressings fell off naturally, and the uninfected- and infected-wounds had both almost completely healed (Fig. 7a), with wound areas of 12.57 ± 2.49% and 13.39 ± 2.37%, respectively (Fig. 7b). In comparison, in the blank control group, the uninfected-wounds retained a little lump and the infected-wounds healed more slowly. Such an excellent wound-healing effect of SCWF-Ag5 might be attributed to the synergistic effects between the anti-bacterial performance of AgNPs and the porous structure of SCWF in combination with the obstruction of ambient bacteria, thereby leading to a dampening of inflammation and facilitating enhanced healing.

### Histological analysis

**Figure 8:**
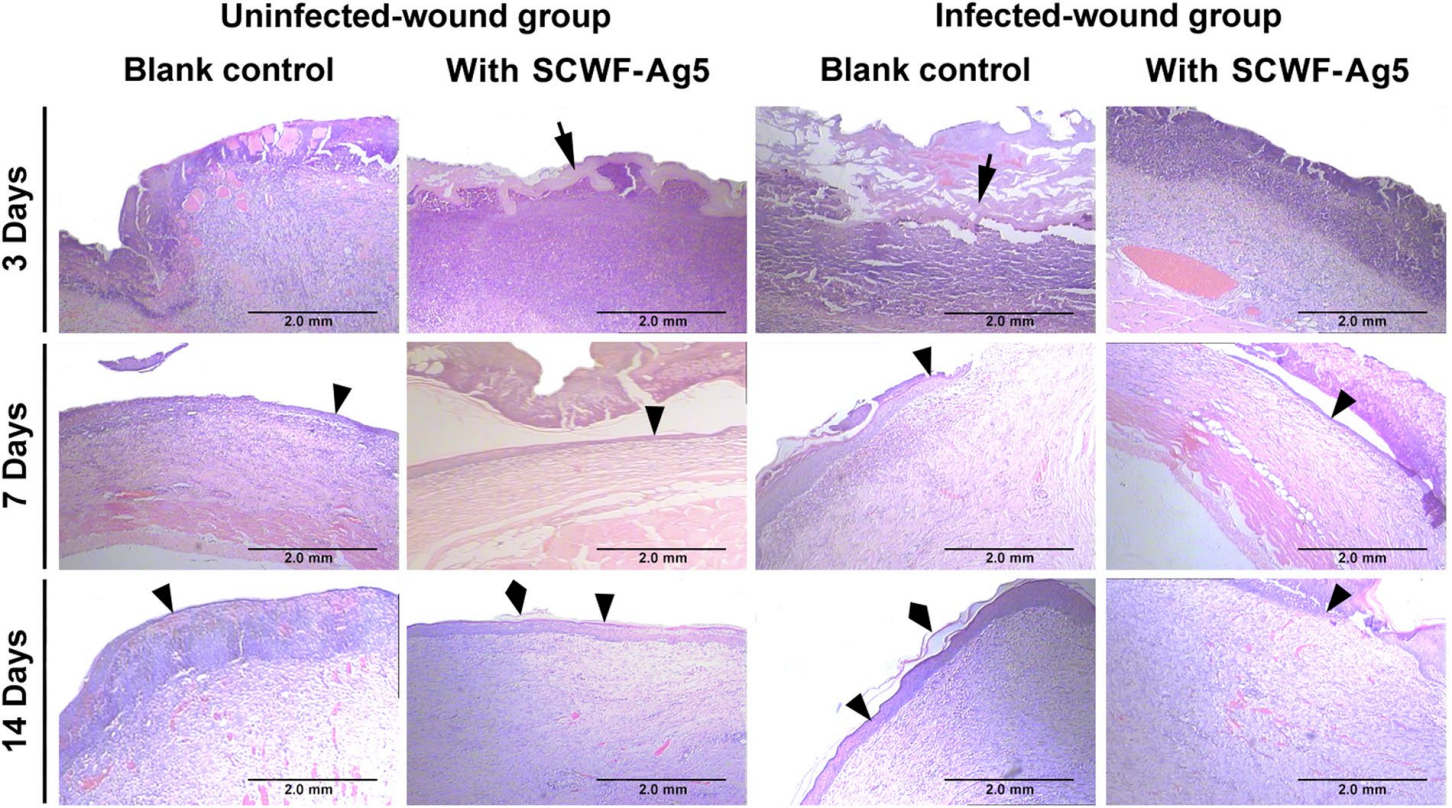
Histological evaluation of skin wound healing at days 3, 7, and 14. Photomicrographs showing sections of skin tissues with H&E staining. Black arrows, triangles, and quadrangles, respectively, indicate SCWF-Ag5, re-epithelialization, and regenerated keratin fibres.

Wound healing is a specific biological process related to tissue growth and regeneration that can be divided into four phases including haemostasis, inflammation, proliferation, and maturation phases^50^. As shown in Fig. 8, a number of inflammatory cells were found to emerge on the wound after 3-day treatments in the uninfected- and infected-wound groups. It could also be seen clearly that the dressings (black arrow) were applied tightly to the wound area. After 3-day treatments, a larger number of inflammatory cells appeared on the untreated infected wound, whereas fewer remained on the infected wound treated with SCWF-Ag5 dressing for 3 days. Some collagen fibres, fibroblasts, and immature glandular cavities appeared on the uninfected- and infected wounds treated with the SCWF-Ag5 dressing for 7 days. The collagen fibres and fibroblasts begin to migrate into the injured area after 7-day treatment with the SCWF-Ag5 dressing, which corresponded to the migration phase of the healing process (black triangles indicate re-epithelialization) and indicated that the SCWF-Ag5 dressing facilitated a quicker wound healing process than that observed in the control groups. SCWF-Ag5 accelerated the regeneration of new epidermal tissue; furthermore, the scab also decreased over time. After 14-day treatments, the uninfected- and infected-wounds without dressing yielded a large quantity of granulation tissue, whereas treatment with the SCWF-Ag5 dressing under the same conditions generated mature epithelial cells that had developed into regenerated keratin fibres (black quadrangle). Thus, it could be concluded that the SCWF-Ag5 dressing exhibited the best wound-healing capability of those tested, especially for infected wounds. Overall, the excellent wound-healing performance exhibited by SCWF-Ag5 over a very short period suggests its suitability for potential clinic applications.

## Conclusions

The protective function of the silkworm cocoons resembles the manner in which the skin protects the human body. When combined with the advantages of fibroin and sericin in the natural cocoon, SCWFs were successfully constructed by partly dissolving silkworm cocoons in a solution of CaCl_2_-ethanol-H_2_O, appearing as transparent elastic films. Then, *in situ* assembly of AgNPs on porous SCWF was effected by using sericin as a reducing agent. The AgNPs were quite homogeneously distributed and exhibited an excellent spherical shape with a particle size ranging around 8 nm. Among the varied SCWFs, SCWF-Ag5 exhibited marked bacteriostatic ability against *S. aureus* and *E. coli*. Following the demonstration of biocompatibility and efficacy, the results of *in vivo* experiments confirmed that infected wounds covered with SCWF-Ag5 exhibited excellent wound healing effects. The application of SCWF-Ag5 expedited the recovery of the full cutaneous thickness of the infected wound, with histology results on day 15 following wound creation revealing less scarring of the wound area. Further confirmation of recovery via histological analysis indicated that the granulation tissue contained fewer inflammatory cells as well as more regenerated keratin fibres, fibroblasts, and capillaries when compared to the wounds of rabbits treated with blank control, demonstrating the potential application of this material in wound treatment. Furthermore, the optical transparency of these dressings facilitated wound observation and their material properties enabled painless wound cleaning during the healing process. Thus, this work provided a simple and “green” means of fabricating a novel SCWF-AgNP composite dressing that possesses considerable potential for clinical application and importance in the field of anti-bacterial materials. This method could greatly decrease the complexity of the fabrication process and reduce manufacturing cost, facilitating ready industrialization.

## Methods

### Materials

The *B. mori* cocoons used in this study were provided by the State Key Laboratory of Silkworm Genome Biology (Southwest University, Chongqing, China). All chemicals were purchased from Taixin Chemical Reagent Company (Chongqing, China) and used without further purification. The mouse L929 fibroblastic cells were provided by the Third Military Medical University. All animal experiments and care were in compliance with institutional ethical use protocols and were approved by the National Center of Animal Science Experimental Teaching (ASET) at the College of Animal Science and Technology (CAST) in the Southwest University of China, in accordance with the college’s “Guide for the Care and Use of Laboratory Animals”.

### Scanning electron microscopy (SEM)

For SEM analysis, thin SCWF samples were snap frozen at −50 °C and subsequently lyophilized to remove their water content. The lyophilized samples were mounted onto sample holders using conductive tape and then sputter-coated with platinum using an E-1045 High Resolution Sputter Coater (Hitachi, Tokyo, Japan) at 30 mA to improve their conductibility. The structural morphology of the lyophilized samples were observed using a JSM-6610 scanning electron microscope (Shimadzu, Kyoto, Japan) using an accelerating voltage of 15 kV.

### Characterization

The mechanical properties of wet SCWF and SCWF-AgNPs including tensile strength, Young’s modulus, and elongation at break were measured on a Shimadzu Autograph AGS-X machine fitted with a 5 N load cell. Briefly, rectangle shaped samples (45 × 20 × 0.7 mm, length × width × thickness) were pulled at a rate of 5 mm/min and elongated to break. The Young’s modulus was obtained by calculating the gradient from 0 to 10% of elongation of the stress-strain curve. The ultimate elongation and the ultimate tensile stress of SCWF and SCWF-AgNPs were obtained according to the fracture data. The wide angle XRD patterns of dried sheets were recorded on an XRD diffractometer (X’Pert PRO XRD, PANalytical, Almelo, The Netherlands; Cu Ka radiation, 40 kV, 40 mA, λ = 0.15418 nm) in the 2 theta angle range of 10°–80°. Thermo-gravimetric analysis of freeze-dried samples (5 mg) was performed using a TG209 instrument (NETZSCH, Selb, Germany) under a nitrogen atmosphere in the temperature range of 40–700 °C at a heating rate of 10 °C min^−1^. The size morphology and distribution of AgNPs in the lyophilized SCWF were characterized using a transmission electron microscope (TEM, JEOL JEM-2010 HT, Tokyo, Japan) using an accelerating voltage of 200 kV. Ultrathin sections were prepared by slicing the samples on a carbon coated copper grid. Fourier transform infrared (FT-IR) measurements were performed using a Bruker alpha FT-IR spectrometer (Karlsruhe, Germany) to analyse the functional groups present in SCWF-AgNPs. Samples were ground and thoroughly mixed with potassium bromide and pelletized. The IR spectra of the pellets were obtained by scanning at the range of 400–4000 cm^−1^.

### Anti-bacterial performance of SCWF-AgNPs

*S. aureus* (ATCC-25923) and *E. coli* (ATCC 25922) were purchased from the Medicine and Biological Laboratories (Chongqing Science University, Chongqing, China) and used following established safety protocols. A bacterium culture solution was prepared (5 g/L NaCl, 5 g/L beef extract, 10 g/L beef peptone, purchased from Aobox, Beijing, China). *S. aureus* and *E. coli* were cultivated at 37 °C in sterilized beef extract broth in a rotary shaker overnight with a speed of 150 rpm and the obtained bacteria suspensions were diluted into various concentrations prior to use.

SCWF and SCWF- AgNPs (SCWF-Ag1–6), were used to test anti-bacterial inhibition halos using a modified Kirby Bauer technique^51^. Briefly, 200 μL bacteria medium (approximately 10^7^–10^8^ CFU/mL; both *S. aureus* and *E. coli* were tested) was dispersed onto a beef extract agar plate (Φ100 × 6 mm). Then, SCWF/SCWF- AgNP disks (approximately Φ10 × 0.7 mm) were placed on the agar plate and incubated for 24 h at 37 °C. After incubation, the bacterial inhibition halos around the SCWF/SCWF- AgNP samples were observed and their diameters were measured.

The bacterial inhibition kinetics toward both *S. aureus* and *E. coli* were tested. For each sample, 0.2 g freeze-dried SCWF/SCWF-AgNPs was immersed in 20 mL germ containing nutrient solution with a bacterial concentration (around 10^7^–10^8^ CFU/mL). Incubation was performed at 37 °C for 24 h in an orbital shaker with a speed of 150 rpm. Bacterial inhibition ratios were calculated by equation^52^. Pure growth broth without bacteria was also tested and served as a control. Samples of the bacterial broth medium were removed at pre-set intervals (200 mL each time) and the OD value of the medium at 600 nm was recorded using a microreader (TECAN infinite M200 PRO, San Jose, CA, USA). The inhibition ratios of SCWF/SCWF-AgNPs were calculated using equation:

$$\mathrm{Inhibition}\,\mathrm{ratio}\,( \% )=100-100\times \frac{{A}_{t}-{A}_{0}}{{A}_{con}-{A}_{0}}$$

where *A*_*0*_ is the OD value of bacterial broth medium before incubation and *A*_*t*_ and *A*_*con*_ are the OD values of SCWF/SCWF-AgNP medium and pure medium (control) after incubation for the designated time, respectively.

### Cytotoxicity assay

The cytotoxicity assay was performed against the L929 murine fibroblast cell line as follows. Each sample with a total weight of 0.2 g was cut into small pieces after sterilization by ultraviolet radiation for 30 min. Subsequently, fragmented sponge samples were immersed in sterilized Dulbecco’s modified Eagle medium (DMEM) to equilibrate. Then, DMEM containing the SCWF samples was transferred to an incubator and cultured for 72 h. L929 cells were seeded in a 96-well plate with a density of 5 × 10^3^ cells/well after incubation in 100 μL DMEM containing 10% foetal bovine serum for 24 h at 37 °C and 5% CO_2_. Then, 10 μL leaching liquor was added to each well. After incubation for various durations (24, 48, and 72 h) at 37 °C and 5% CO_2_, cell proliferation was assessed using a determined MTT enzyme assay with 490 nm absorbance detection (Multiskan MK3, Thermo Fisher Scientific). Each analysis was replicated 5 times.

Each thin slice of SCWFs and SCWF-AgNPs was then immersed in the DMEM on the surface of the L929 cells in a 24-well plate for 24 h. An equal amount of SCWF was used as a control. The thin slices were then gently removed, and the media were replaced with 100 μL of fresh DMEM for further treatments. The media were then observed and photographed using a microscope (Nikon). The viability of the cells was examined using a YEASEN^®^ Calcein-AM/PI Double Stain Kit following the manufacturer’s protocol, with imaging under a fluorescence microscope.

### *In vivo* infected wound healing

As *in vivo* experiments are indispensable for evaluating the actual wound-healing effects of potential wound dressings, we evaluated the infected wound healing efficacy of SCWF-AgNPs using a New Zealand rabbit model, as previously noted, all animal experiments and care were in compliance with institutional ethical use protocols. Adult male New Zealand rabbits (2.3 ± 0.1 kg) were subjected to a dorsal operation under general anaesthesia. To obtain credible evaluation of the wound dressing anti-bacterial activity to accelerate wound healing, all groups were provided with uniform degrees of tissue injury wounds as follows. Briefly, four full-thickness cutaneous wounds (diameter 20 mm) were generated on the dorsum of each rabbit (n = 12). The four wounds were divided randomly into two groups, the uninfected- and infected-wound groups. For the uninfected wound group, one wound was covered with an SCWF-AgNP and the other wound was left without dressing as a blank control. For the infected wound group, a 200 μL aliquot of *S. aureus* suspension (1.0 × 10^8^ CFU mL^−1^) was applied to two wound areas, whereupon the wounds became severely infected after 24 h. Then, as for the uninfected groups, one wound was covered with an SCWF-AgNP and the other wound was left without dressing as a blank control. All of the wounds were photographed. After positioning the SCWFs, gauze and crepe bandages were used to secure the dressings. Rabbits were housed individually and monitored daily for food and water consumption. To evaluate the rate of healing, the area of the wound was determined at several time points (3, 7, 14, and 21 days). After each time point, three animals were euthanized and skin samples were collected from the various wounds. Each sample was preserved in 10% formalin.

### Histological examination

The formalin-fixed tissue samples were dehydrated in an ascending series of alcohol, cleared with xylene, and embedded in paraffin wax. Paraffin sections (4 mm thick) were then cut and dried in an oven overnight. The sections were then stained in haematoxylin and eosin (H&E staining) and visualized with 3,30-diaminobenzidine, dehydrated, and mounted for viewing under an Olympus BX51 microscope (Tokyo, Japan).

### Statistical analysis

Statistical analysis was performed using SPSS version 19 (Chicago, IL, USA). The data were analysed statistically using a one-way analysis of variance (ANOVA) with Duncan’s multiple range tests. The data represent the means ± standard deviation (SD) of each group. In both graphs, asterisks represent the P < 0.05 level, indicating that the means were statistically significant in comparison with the control group.

## Electronic supplementary material

Supporting Information
