# Author Correction: *In situ* assembly of Ag nanoparticles (AgNPs) on porous silkworm cocoon-based wound film: enhanced antimicrobial and wound healing activity

**DOI:** 10.1038/s41598-018-19257-6

**Published:** 2018-01-22

**Authors:** Kun Yu, Fei Lu, Qing Li, Honglei Chen, Bitao Lu, Jiawei Liu, Zhiquan Li, Fangying Dai, Dayang Wu, Guangqian Lan

**Affiliations:** 1 College of Textile and Garments, Southwest University, Chongqing, 400715 China; 2 Chongqing Engineering Research Center of Biomaterial Fiber and Modern Textile, Chongqing, 400715 China; 3 The Ninth People’s Hospital of Chongqing, Chongqing, 400700 China

Correction to: *Scientific Reports* 10.1038/s41598-017-02270-6, published online 18 May 2017

The original version of this Article contained a typographical error in the title.

“*In situ* assembly of Ag nanoparticles (AgNPs) on porous silkworm cocoon-based would film: enhanced antimicrobial and wound healing activity”

now reads:

“*In situ* assembly of Ag nanoparticles (AgNPs) on porous silkworm cocoon-based wound film: enhanced antimicrobial and wound healing activity”

In addition, there were errors in Figure 2 and Figure 6. In Figure 2c, the micrograph for SCWF-Ag6 (right bottom) duplicated the micrograph for SCWF-Ag5 (right middle) and has now been replaced. In Figure 6A, panel c’ (SCWF-Ag6) duplicated panel a’ (SCWF) and has now been replaced.

**Figure 2:**
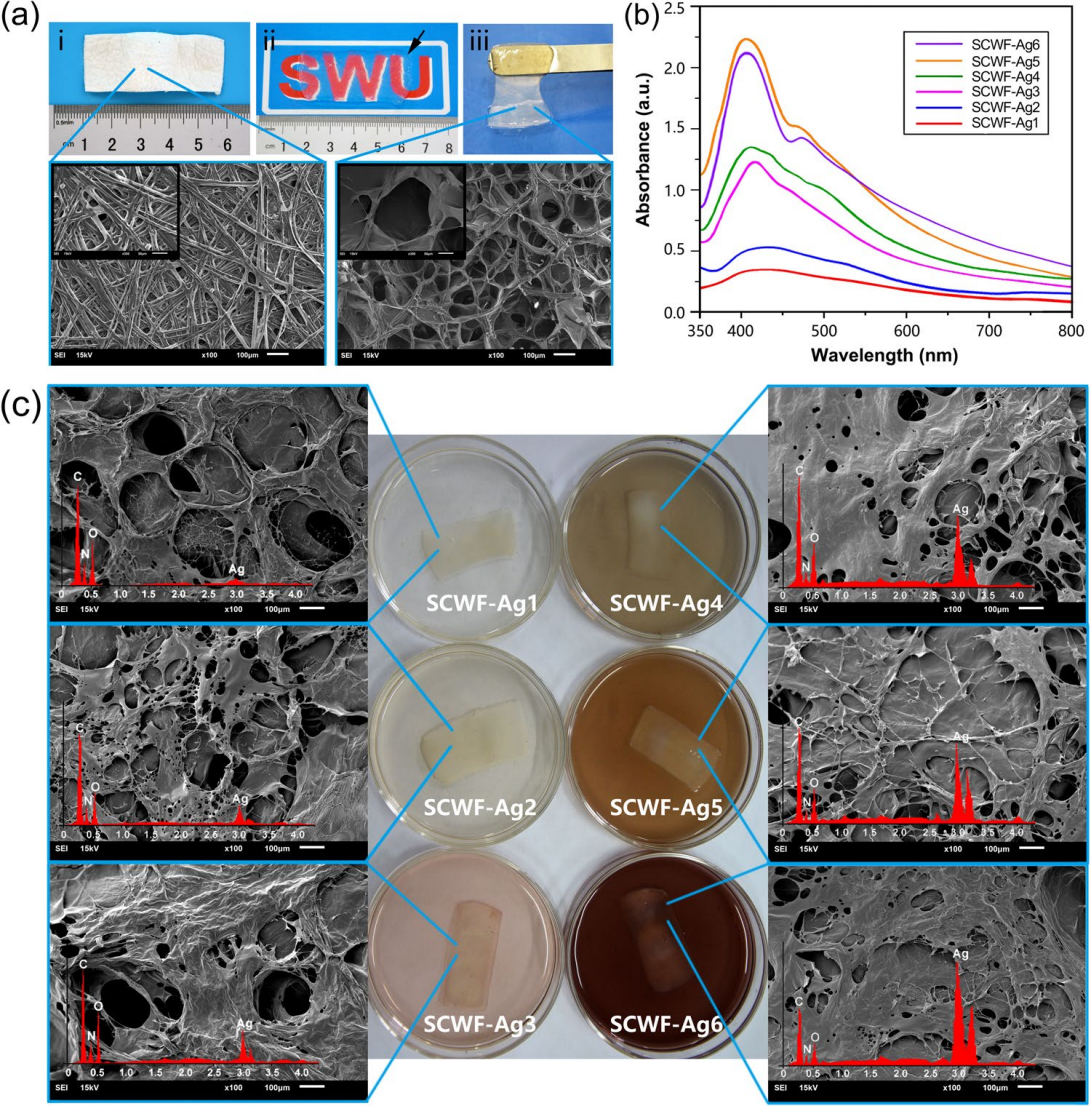
(**a**) Natural *Bombyx mori* cocoons (i); SCWF optical clarity and size (ii), wherein SCWF (black arrow) was placed on a piece of paper with one “SWU” symbol underneath. The gel (balanced on a metal spatula) was sufficiently elastic and flexible for easy handling (iii). The fibroin and sericin further dissolved to form a network that readily aggregated into transparent films, as shown in the scanning electron micrograph of lyophilized SCWF. (**b**) UV-vis absorption spectra of leaching aqueous dispersions of SCWF-Ag1–6 in deionized water. (**c**) Scanning electron micrographs of lyophilized SCWF-Ag1–6 with red curves of EDX and photographs of the respective SCWFs immersed in different concentration of AgNO3 aqueous solution after 4 h.

**Figure 6:**
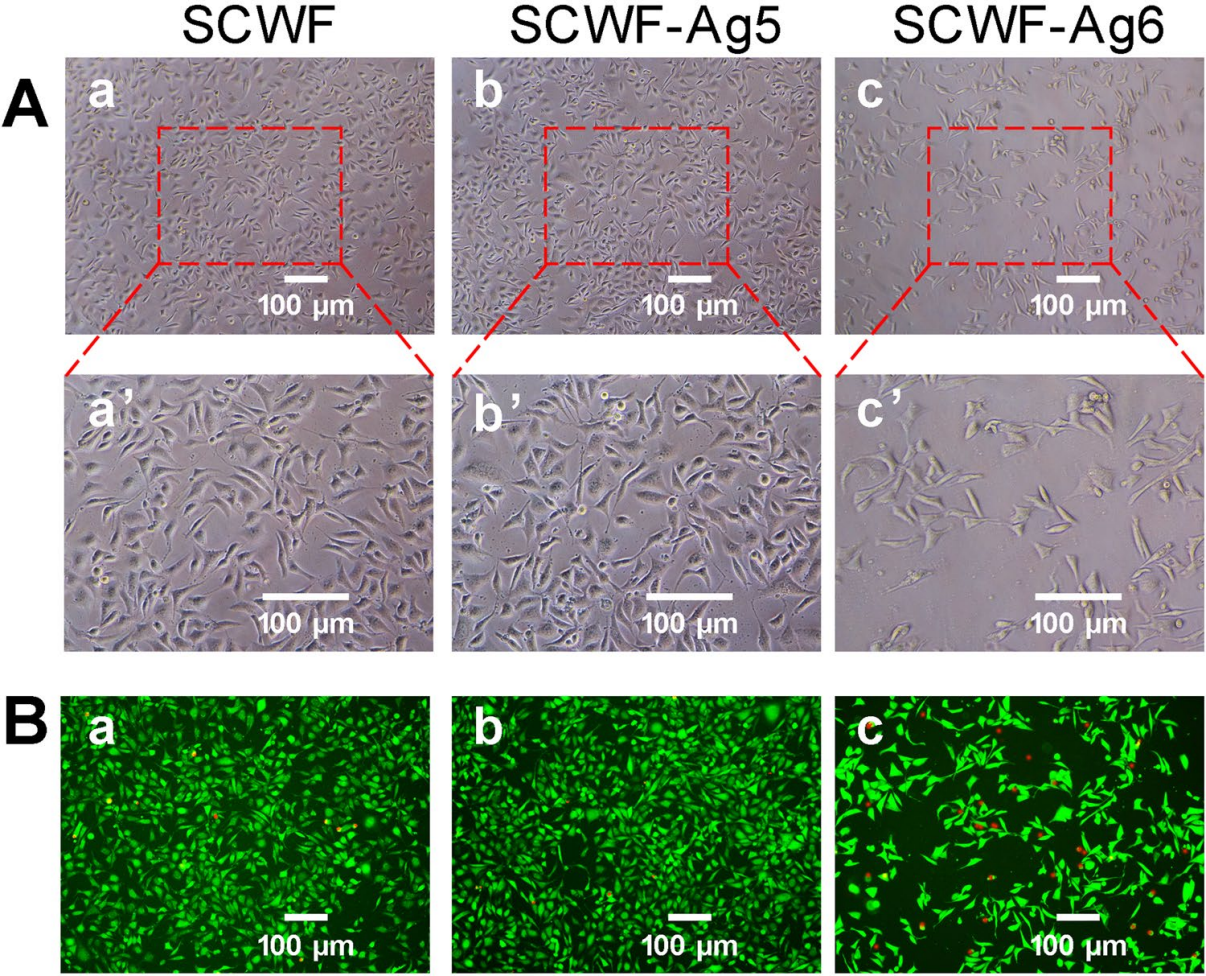
(**A**) Growth observations of L929 cells treated with SCWF (a,a’), SCWF-Ag5 (b,b’), and SCWF-Ag6 (c,c’). (**B**) Calcein-AM/PI Double Stain Kit assay of L929 cells upon treatment with SCWF (a), SCWF-Ag5 (b) and SCWF-Ag6 (c). Live cells are stained by Calcein AM dye and produce an intense uniform green fluorescence (ex/em ~495 nm/~515 nm). Dead cells are stained by Calcein PI dye and emit bright red fluorescence (ex/em ~495 nm/~635 nm). The scale bar represents 100 μm.

These errors have now been corrected in the PDF and HTML versions of the Article

