# Supplementary material for: In situ assembly of Ag nanoparticles (AgNPs) on porous silkworm cocoon-based wound film: enhanced antimicrobial and wound healing activity
Source: Sci Rep. 2017 May 18;7:2107. doi: 10.1038/s41598-017-02270-6 (PMC5437089; doi:10.1038/s41598-017-02270-6)
Supplement: Supplementary file 1 — Supporting Information [file 41598_2017_2270_MOESM1_ESM.pdf]

Supporting Information

In situ assembly of Ag nanoparticles (AgNPs) on porous silkworm cocoon-based wound film: enhanced antimicrobial and wound healing activity

Kun Yu[a,1], Fei Lu[a,b,1], Qing Li[a], Honglei Chen[a], Bitao Lu[a], Jiawei Liu[a], Zhiquan Li[c], Fangying Dai[a,b], Dayang Wu[a,b], Guangqian Lan[a,b,*]

(a College of Textile and Garments, Southwest University, Chongqing 400715, China)

(b Chongqing Engineering Research Center of Biomaterial Fiber and Modern Textile, Chongqing 400715, China)

(c The Ninth People's Hospital of Chongqing, Chongqing 400700, China)

* Corresponding author. E-mail: 30353930@qq.com. Tel: +86-13594005200.

1 These authors contributed equally to this work.

KEYWORDS: Silkworm cocoon, Silver nanoparticles**,** Infected wound healing, Silk fibroin, Antibacterial performance

Figure S1

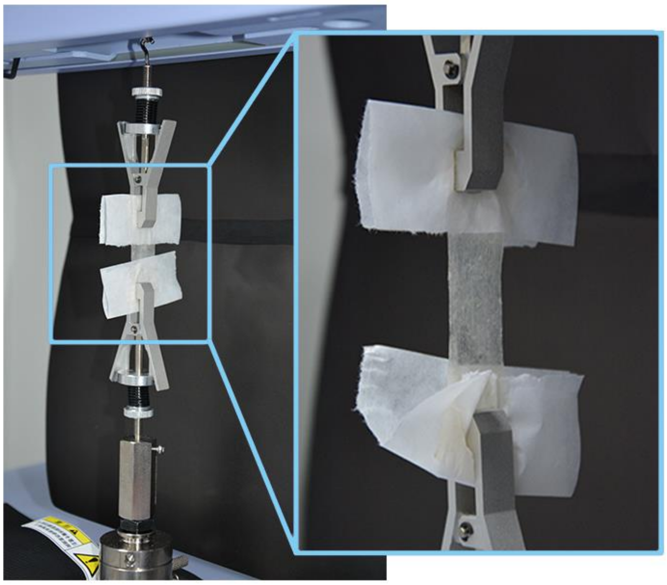

Figure S1. Images of the setup used for stretching the sample and measuring its tensile mechanical properties. A layer of paper is provided between the fixture and sample, to prevent the sample fracturing in the fixture.

Figure S2

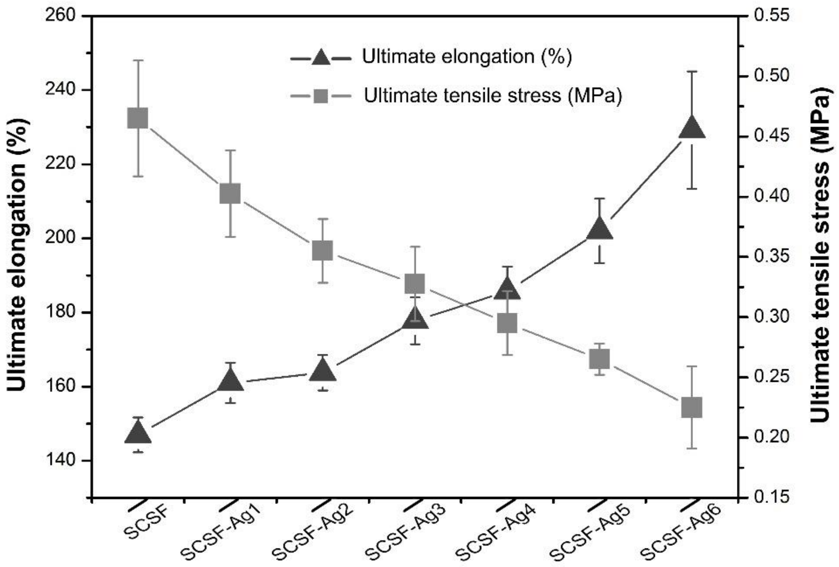

Figure S2. Ultimate elongation and ultimate tensile stress. The error bars denote the standard error of the mean (n = 10). The ultimate elongation values all exceeded 140%. As the AgNPs content increased, the

ultimate elongation increased from 147.0 ± 4.7% in SCWF to 229.3 ± 15.8% in SCWF-Ag6 (Figure S2). The Ag^+ that consume a lot of sericin result in a further decrease of the stress, and thus more elasticity.

Figure S3

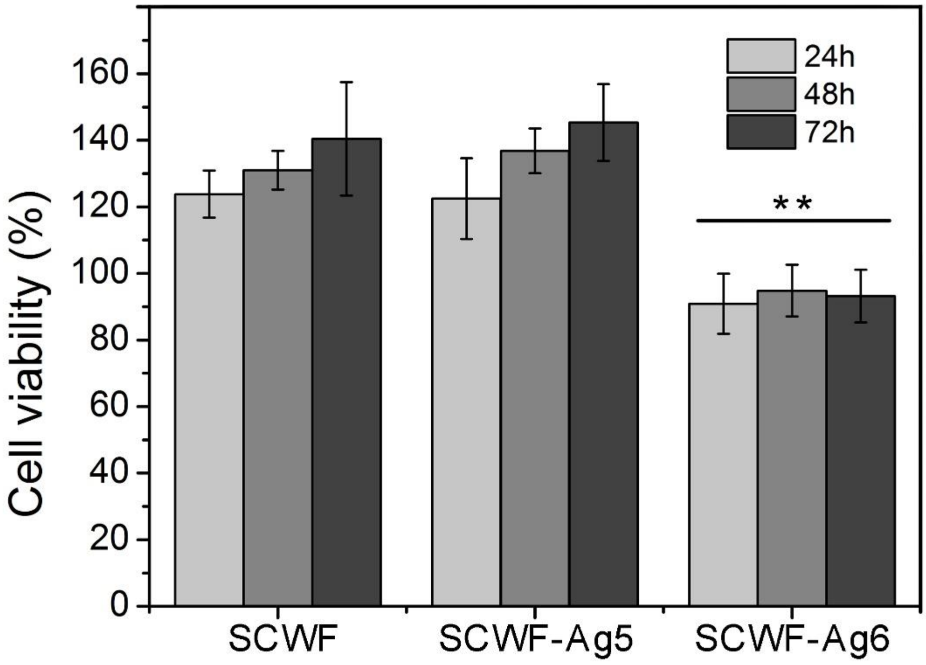

Figure S3. Viability of L929 murine fibroblast cells after 24, 48, and 72 h of contact with leaching liquors obtained from SCWF and SCWF-Ag5. The leaching liquor obtained from SCWF-Ag5 revealed increased cell viability compared to the SCWF control. The error bars denote the standard error of the mean (n = 3, **P < 0.01).
